# Selective increase in subtelomeric DNA methylation: an epigenetic biomarker for malignant glioma

**DOI:** 10.1186/s13148-015-0140-y

**Published:** 2015-10-07

**Authors:** Samrat Roy Choudhury, Yi Cui, Jacob R. Milton, Jian Li, Joseph Irudayaraj

**Affiliations:** Department of Biological Engineering, Center for Cancer Research, Purdue University, West Lafayette, IN 47906 USA; Department of Biological Sciences, Purdue University, West Lafayette, IN 47906 USA; Department of Neurosurgery, Xiangya Hospital, Central South University, Changsha, Hunan 410008 China

**Keywords:** Subtelomeric DNA methylation, Telomere length, Glioma, Epigenetic biomarker

## Abstract

**Background:**

Subtelomeric regions dynamically change their epigenetic pattern during development and progression of several malignancies and degenerative disorders. However, DNA methylation of human subtelomeres and their correlation to telomere length (TL) remain undetermined in glioma.

**Results:**

Herein, we report on the selective changes in subtelomeric DNA methylation at the end of five chromosomes (Chr.) (7q, 8q. 18p, 21q, and XpYp) and ascertain their correlation with TL in patients with glioma. Subtelomeric methylation level was invariably higher in glioma patients compared to the control group, irrespective of their age and tumor grade. In particular, a significant increase in methylation was observed at the subtelomeric CpG sites of Chr. 8q, 21q, and XpYp in tissues, obtained from the brain tumor of glioma patients. In contrast, no significant change in methylation was observed at the subtelomere of Chr. 7q and 18p. Selective changes in the subtelomeric methylation level, however, did not show any significant correlation to the global TL. This observed phenomenon was validated in vitro by inducing demethylation in a glioblastoma cell line (SF-767) using 5-azacytidine (AZA) treatment. AZA treatment caused significant changes in the subtelomeric methylation pattern but did not alter the TL, which supports our hypothesis.

**Conclusions:**

DNA methylation level dramatically increased at the subtelomere of Chr.8q, 21q, and XpYp in malignant glioma, which could be used as an early epigenetic diagnostic biomarker of the disease. Alterations in subtelomeric methylation, however, have no effects on the TL.

**Electronic supplementary material:**

The online version of this article (doi:10.1186/s13148-015-0140-y) contains supplementary material, which is available to authorized users.

## Background

Malignant glioma is one of the most common, locally invasive and aggressive form of primary brain tumors that predominantly manifests in adults but is also frequently reported in infants [1, 2]. In its most aggressive manifestation, glioblastoma multiforme, the median survival of patients is approximated to a year, and survivors over 3 years are referred to as the “long-term survivors” [3, 4]. Despite the accessibility to multimodal therapeutics including surgery, chemotherapy and radiation therapy, the overall therapeutic benefit against glioma remains unsatisfactory [5]. One of the major focuses in clinical research hence has been aimed to identify the potential early stage signatures imprinted at the genomic or epigenomic loci of glioma [6]. The modified epigenetic marks such as DNA hypermethylation or hypomethylation of different gene promoters are already reported from several clinical studies in conjunction with glioma patients [7–9]. In particular, the occurrence of hypermethylation at the *MGMT* (O^6^-methylguanine DNA-methyltransferase) promoter, located at Chr. 10q26, has been identified as one of the significant molecular markers of glioma [10]. The acquired methylation at the *MGMT* promoter results in silencing of the gene, which has been associated with prolonged survival in glioma patients, due to the enhanced susceptibility of tumor cells to the chemotherapeutic agents such as temozolomide [11, 12]. Besides *MGMT*, hypermethylation at the promoter of several tumor suppressors, apoptotic, or Wnt-signaling pathway involved genes and hypomethylation of normally silenced genes such as CD133, MMP9, or IL8 have also been identified among the glioma patients [13–15].

Telomeres positioned at the ends of chromosomes are specialized nucleoprotein complexes containing multiple arrays of duplex TTAGGG repeats and a complex of telomere repeat binding proteins [16, 17]. Telomeres prevent degradation of chromosome (Chr.) ends and their exposure to unnecessary DNA repair activities and recombinations [16]. Telomere adjacent subtelomeric domains are also considered to be a part of heterochromatic regions [18], which are enriched with the promoters of several genes, segmental duplications, satellite sequences, or telomere-like interstitial TTAGGG repeat sequences. In addition, subtelomeres harbor highly repetitive CpG dinucleotides, which are otherwise absent in telomeres and are prone to epigenetic changes, mainly the DNA methylation. Aberrant epigenetic alterations at the subtelomeric CpGs due to induced methylation or hydroxymethylation have been reported to influence several biological pathways or induce cellular reprogramming; the detailed mechanisms of which are yet to be understood [19]. The random epigenetic modifications at the subtelomeric repeat elements or embedded gene promoters are also speculated to influence telomere length (TL), which is otherwise tightly regulated [18, 20]. Several studies have suggested that dense cytosine methylation at the subtelomeric CpG islands may be involved in silencing the noncoding RNA referred to as the telomere repeat-containing RNA (TERRA), a potential negative regulator of telomerase. Methylation mediated transcriptional repression of the TERRA promoter, embedded in the subtelomeric regions may facilitate telomerase access resulting in telomere lengthening in telomerase positive tumor cells [21–23]. In contrast, triple negative knockdown of Ten eleven translocation (TET) methylcytosine deoxygenase proteins in mouse embryonic stem cells, which caused depletion of methylated cytosines in the chromosomal ends, is reported to result in elongated telomere [24]. Moreover, some recent reports have demonstrated the correlation between the subtelomeric DNA methylation to the development of several degenerative disorders and some sporadic malignancies [25–27]. In summary, studies show that DNA methylation of subtelomeric regions plays a crucial role in the regulation of TL [28].

Acquired hypermethylation at the promoter of several neural and proneural genes have also been identified during the occurrence and progression of glioma [7, 8, 29]. However, to the best of our knowledge, changes in the DNA methylation of human subtelomeres and their correlation to TL remain undetermined in glioma. Herein, we report the occurrence of selective increase in methylation at the subtelomeric CpG sites of specific chromosomes in a cohort of glioma patients, which could serve as potential epigenetic biomarker for the disease. In addition, we have evaluated the correlation between the changes in DNA methylation at the subtelomeric loci to the global TL.

## Methods

### Cell and tissue samples

Tissue samples were collected from the non-glioma patients (*n* = 13), who were subjected to surgeries for severe accidental injuries in the brain. In contrast, tissue samples from the glioma patients (*n* = 15) were obtained from tumor tissues, resected after neurosurgery. Tumor grade was determined by a certified pathologist according to the 2007 World Health Organization (WHO) Classification of Tumors of the Central Nervous System. Tissue samples were frozen directly in liquid nitrogen and stored at −80 °C. Glioma tissue biopsies were collected from patients of different demographic and clinicopathologic features from the Xiangya Hospital of Central South University at Hunan province, China. Informed consent was obtained from all of the involved patients. This study was approved by the Ethical Committee of Xiangya Hospital of Central South University. In addition to patient tissue samples, we have also used a standard glioblastoma cell line SF767 in the present study for in vitro cross validation of the observed correlation between change in subtelomeric DNA methylation and TL.

### Extraction of genomic DNA

Genomic DNA from control or tumor tissues were isolated in cell lysis buffer (100 mM Tris-HCl, 5 mM EDTA, 0.2 % SDS, 200 mM NaCl, 0.667 μg/μl Proteinase K) at 55 °C overnight. In the second day, samples were added with an equal volume of phenol:chloroform:isoamyl alcohol (25:24:1 saturated with 10 mM Tris, 1 mM EDTA), mixed evenly and centrifuged for 5 min at 14,000*g*. The supernatant was then transferred into a new tube and precipitated with equal volume of 100 % isopropanol. The genomic DNA was recovered and dissolved with 10 mM Tris-HCl. Genomic DNA samples were further sonicated into 500 bp by Misonix sonicator 3000. The concentration of sonicated DNA was then determined by a Nano-Drop 1000 system (Thermo Scientific, USA). DNA integrity and RNA contamination were assessed by gel electrophoresis.

### Determination of global change in DNA methylation

Genome-wide methylation level (5-mC %) in both the control and glioma patients were determined with MethylFash Methylated DNA Quantification Kit (Epigentek, NY, USA) according to manufacturer’s protocol. Briefly, genomic DNA was extracted from the tissue samples (as mentioned above), and 5-mC% was determined out of 100 ng of the DNA in triplicate. A methylated and unmethylated DNA (20 μg/ml each) sample was used as positive and negative control, respectively, and for determining the relative methylation level for each patient in the tested groups.

### Bisulfite conversion of genomic DNA

Genomic DNA isolated from the tissue or cell samples were bisulfite converted using the EZ DNA methylation kit (Zymo Research, CA, USA). Briefly, 500 ng of DNA sample was mixed with 5 μl of M-dilution buffer in the total volume of 50 μl and incubated at 37 °C for 15 min. One hundred microliters of Cytosine to Thymidine (CT) conversion agent was then added to the samples and incubated further at 50 °C for 14–16 h. Modified DNA was purified using Zymo spin columns along with the provided buffers in the kit. The bisulfite converted DNA samples were then eluted in 10 μl of elution buffer and evaluated for DNA concentration.

### Methylation specific PCR

Subtelomeric methylation level was primarily determined in triplicate using methylation specific polymerase chain reaction (methylation specific PCR (MSP)) and expressed as the methylation ratio (%). Methylation and unmethylation ratio from the band intensities of the gel images were calculated, as described in the previous study. For this study, methylation ratio over 50 % was considered hypermethylated, and less than 50 % was considered hypomethylated [27]. MSP primers were selected against the subtelomeric regions of five different chromosomes namely 7q, 8q, 18p, 21q, and XpYp, reported elsewhere [27]. MSP was carried out using the Pyromark PCR kit (QIAGEN, CA, USA) according to manufacturer’s specification. Briefly, 20 ng of bisulfite converted DNA was mixed with 1 X Pyromark PCR master mix, 1× coral load solution, 1 μM MgCl_2_, 0.2 μM primer (each), and adjusted with water to constitute a reaction volume 25 μl. PCR reaction conditions were retained as in the original report. PCR products were resolved in 2 % agarose gels, and the band intensities were analyzed with the ImageJ software (National Institutes of Health, Bethesda, MD, USA; http://rsb.info.nih.gov/ij/). Amplicon size for all the observed subtelomeric regions were less than 500 bp. A 100-bp DNA ladder was used in each case for convenience.

### Bisulfite PCR and pyrosequencing

Bisulfite PCR (BSP) and pyrosequencing primers were designed against the region that flanks over the MSP amplicons, to quantitatively determine the percentage of methylation difference at the target CpG sites between the control and glioma patients using the Pyromark Assay Design tool (Sw2, QIAGEN, CA, USA). PCR primers, reaction conditions, sequencing primers, and target genomic sequences are summarized in Additional file 1: Table S1 and S2.

### Telomere length measurement

A quantitative real-time PCR (qPCR) was performed in MicroAmp optical 96-well reaction plates using StepOnePlus Real-Time PCR Systems (v 2.0, Applied Biosystems) to determine the TL of the genomic DNA from both the cell line or patient tissue samples. All qPCR assays were run in triplicate with a total reaction volume of 20 μl comprising of 1× Power SYBR Green PCR Master Mix (Life Technologies, USA), 200 nM of both the forward (CGGTTTGTTTGGGTTTGGGTTTGGGTTTGGGTTTGGGTT) and reverse (TTGCCTTACCCTTACCCTTACCCTTACCCTTACCCT) primers (Integrated DNA technologies, IA, USA), and 2 μl of target DNA solution adjusted to the total reaction volume. TL length was determined, followed by the existing standard guidelines [30, 31] with slight modifications. Initially, a standard curve was prepared by plotting the cycle threshold (*C*_*T*_) values against six different dilutions (500, 250, 100, 50, 5, and 0.5 pg, respectively) of a telomere oligomer standard (TTAGGG repeated for 14 times; IDT, USA), amplified with the aforementioned primer set. For the test samples, a uniform DNA concentration (20 ng) was maintained by adjusting the volume with PUC-19 plasmid instead of PBR3222, as mentioned in the previous report [31]. Each tube hence contained equal amount of DNA but differentially concentrated telomeric DNA. The *C*_*T*_ values, obtained from different test samples were then fitted to the telomere standard curve to determine the TL (T) expressed as [log (kb)] / reaction. In addition, the *C*_*T*_ value from the single copy gene 36B4 was used to determine the number of diploid genome (S), and the final TL was expressed in terms of total length of telomere (in kilobase) per human diploid genome (T/S). T/S values were occasionally represented in kilobase (adjusted with the formula) [32], when required to mention the absolute value of TL.

### Cell line studies and treatment with 5-azacytidine

Human glioblastoma cell line SF-767 was cultured in Iscove’s modified Dulbecco’s medium (IMDM) supplemented with 10 % fetal bovine serum, 1 % antibiotics, and 1 % glutamate at 37 °C. After the cells reached 80 % confluence, 3 μM 5-azacytidine (AZA) was prepared with fresh medium and the cells were treated for 72 h and the cell viability was monitored. Cells treated with DMSO were used as a negative control.

### Statistical analysis

To determine the difference between average mean methylation levels and to analyze the independence of CpG methylation between the control and glioma patient groups, a two tailed Mann–Whitney *U* test was performed. The coefficient of variation (Cv) between different round of experiments for MSP and pyrosequencing analyses are mentioned in the supplementary note of SI. Pearson’s correlation coefficient (R) was used to evaluate the strength of association between TL (T/S) and levels of DNA methylation at different subtelomeric CpG sites from the selected chromosomes. Multivariate linear regression analysis was performed to adjust the potential confounders and test of interaction. A *p* value of <0.05 was considered statistically significant for all the obtained data.

## Results

### Correlation between patient characteristics and subtelomeric methylation

The demographic and clinicopathological features of patients, enrolled in this study, are summarized in Table 1. Since the sample size of the present study was relatively small, we cannot predict the statistical correlation between the occurrence of glioma in relation to age or gender. However, we have assessed the difference in subtelomeric methylation level (%) at five chromosomes between the control (non to –glioma) and glioma patients for three age groups (<30 y, 30–60 y, and >60 y) (Additional file 1: Figure S1 and S2). In non-glioma patients, subtelomeric methylation levels were either gradually reduced over the ages, such as in Chr. 7q, 8q, and XpYp or remained almost unchanged such as in Chr.18p and 21q. In contrast, we observed high methylation level at the subtelomeric CpG sites among glioma patients, at all age groups. Although the methylation level did not show any significant alteration among the groups categorized on the basis of clinicopathological features such as tumor grade or tissue position but were invariably higher in glioma patients compared to the control patients (data not shown). In summary, subtelomeric methylation levels were consistently higher in glioma patients at all age groups, addressed in this study, and the change in methylation level can be detected even at the early stage of glioma (grade-II). These features propose the selective subtelomeric sites to be considered as the key biomarkers of glioma.

**Table 1:** Demographic and clinicopathologic characteristics of control and glioma patient cases

Variables	Control (*n* = 13)	Glioma (*n* = 15)
Gender		
Male	12 (92.3 %)	8 (53.34 %)
Female	1 (7.69 %)	7 (46.67 %)
Age (year)		
< 30	2 (15.38 %)	5 (33.34 %)
30–60	7 (53.84 %)	8 (53.34 %)
> 60	4 (30.76 %)	2 (13.34 %)
Smoking (in last 5 year)		
Smoker	2 (15.38 %)	2 (13.34 %)
Non-smoker	11 (84.61 %)	13 (86.67 %)
Education level		
High school and above	5 (38.46 %)	8 (53.34 %)
Below high school	8 (61.53 %)	7 (46.67 %)
Income level (annual)		
< $5000	9 (69.23 %)	11 (73.34 %)
$5000–$10,000	1 (7.68 %)	2 (13.34 %)
> $10,000	3 (23.07 %)	2 (13.34 %)
Biopsy tissue position^a^		
Frontal	7 (53.84 %)	7 (46.67 %)
Temporal	5 (38.46 %)	4 (26.67 %)
Parietal	0	2 (13.34 %)
Occipital	1 (7.69 %)	3 (20 %)
Others^b^	0	2 (13.34 %)
WHO tumor grade		
Low-grade glioma (II)	NA	5 (33.34 %)
Malignant glioma (III)	NA	6 (40 %)
Glioblastoma (IV)	NA	4 (26.67 %)
Post-surgery treatment^c^		
Radiotherapy	NA	1.8 Gy/d (total dose, 50–60 Gy, including 30–36 Gy for brain/spine and 20 Gy for tumor local boost
Chemotherapy	NA	Oral administration of temozolomide (150 mg/m^2^/d) for first 5 days in a 28-day cycle (total 8 cycles of treatment)

*NA* not applicable

^a^Brain biopsy tissues from three glioma patients were obtained from more than one location. Example includes one of the patients had tumor growth in both frontal and temporal lobe, while two other patients had tumor growth in both parietal and occipital lobe

^b^Other positions of biopsy tissue samples include regions such as the fourth ventricle and cerebellum

^c^Individuals that belong to both the cohort of normal and glioma patients were considered based on the criteria that they had no history of other chronic diseases or known long-term exposure to any drugs

### Global methylation vs subtelomeric methylation level

**Fig. 1:**
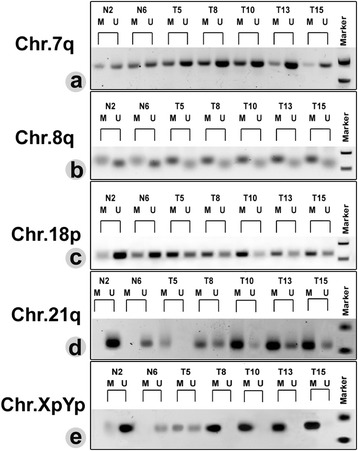
A representative result of methylation specific PCR (MSP) in the subtelomeric region on Chr. 7q (**a**), 8q (**b**), 18p (**c**), 21q (**d**), and XpYp (**e**). M and U refers to the amplification from the methylation- and unmethylation-specific primers respectively. Representative patients were identified by numbers. Chr. 7q subtelomere was partially methylated, and methylation level did not change significantly between the control and glioma patients. Chr.8q subtelomere was also partially methylated, but the methylation ratio increased significantly among the glioma patients. Chr. 18p subtelomere in majority of the non-glioma patients was noticeably amplified with U primers. However, distinct M-specific amplification was observed among the glioma patients. In contrast, subtelomeres of Chr. 21q and XpYp were mostly unmethylated in the non-glioma patients but showed significant increase in methylation level among the glioma patients

**Fig. 2:**
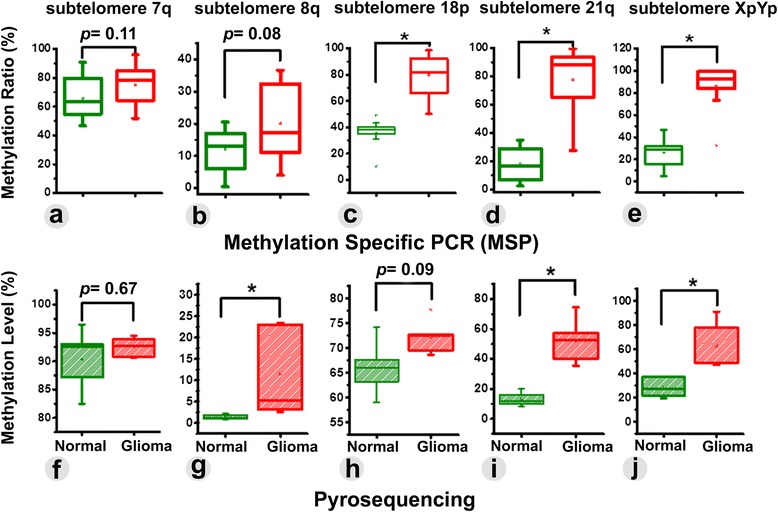
The difference in subtelomeric DNA methylation ratio, as obtained from MSP between control and glioma patients were found significant for Chr. 18p (**c**), 21q (**d**), and XpYp (**e**). In contrast, insignificant alteration in methylation ratio was observed at the subtelomere of Chr. 7q (**a**) and 8q (**b**). The change in subtelomeric methylation level was cross-validated with pyrosequencing analysis. In comparison to MSP, pyrosequencing data revealed significant change in average methylation level at Chr. 8q (**g**), 21q (**i**), and XpYp (**j**) but insignificant methylation alteration for Chr. 7q (f) and 18p (**h**)

The percentage of genome-wide 5-methylcytosine (5-mC %) in the cohort of control patients was in the range between 0.89 and 1.3 %, with the median value of 1.11 %. The 5-mC % assessed in the samples from glioma patients was distributed between 0.9 and 1.4 %, with a median value of 1.2 %. However, we did not find any statistically relevant difference (*p* = 0.81) in the mean 5-mC level between the non-glioma (1.08 ± 0.28 %) and glioma (1.19 ± 0.39 %) patients (Additional file 1: Figure S3). On the contrary, much interesting and diverse results were obtained from the methylation pattern at the observed subtelomeric regions. The subtelomere of Chr. 7q was found to be partially methylated in both the groups, since the amplification was observed against both the methylation (M)- and unmethylation (U)-specific primers. However, the methylation level for both the groups was determined to be over 50 % (hypermethylated). The mean methylation level in non-glioma individuals (65.62 ± 10.10 %) did not significantly differ from the glioma patients (75.05 ± 13.38 %) (Figs.1a and 2a). The subtelomere of Chr. 8q was also partially methylated but showed reduced methylation level (<50 %) in both the groups. The difference in the mean methylation of glioma (16.58 ± 14.1 %) and non-glioma (12.03 ± 6.70 %) groups was not significantly different (Figs.1b and 2b). The subtelomeric domain of Chr. 18p was partially methylated with the difference in mean methylation between the non-glioma to glioma patients were 35.39 ± 10.10 % and 79.68 ± 14.25 %, respectively, which exhibit a statistically significant difference (Figs.1c and 2c). In contrast, the CpG sites at the subtelomeres of Chr. 21q and XpYp showed a general trend in hypomethylation (<50 %) among the non-glioma patients and hypermethylation (>50 %) among the glioma patients. The mean methylation level in non-glioma to glioma patients varied from 17.92 ± 11.25 % to 77.70 ± 21.34 % in chromosome 21q (Figs.1d and 2d) and 26.10 ± 13.80 % to 86.23 ± 19.60 % in chromosome XpYp (Figs.1e and 2e). The mean differences in methylation level percentage between the groups for these two chromosomes hence can be considered significant.

### CpG specific changes in methylation at the subtelomeres

**Fig. 3:**
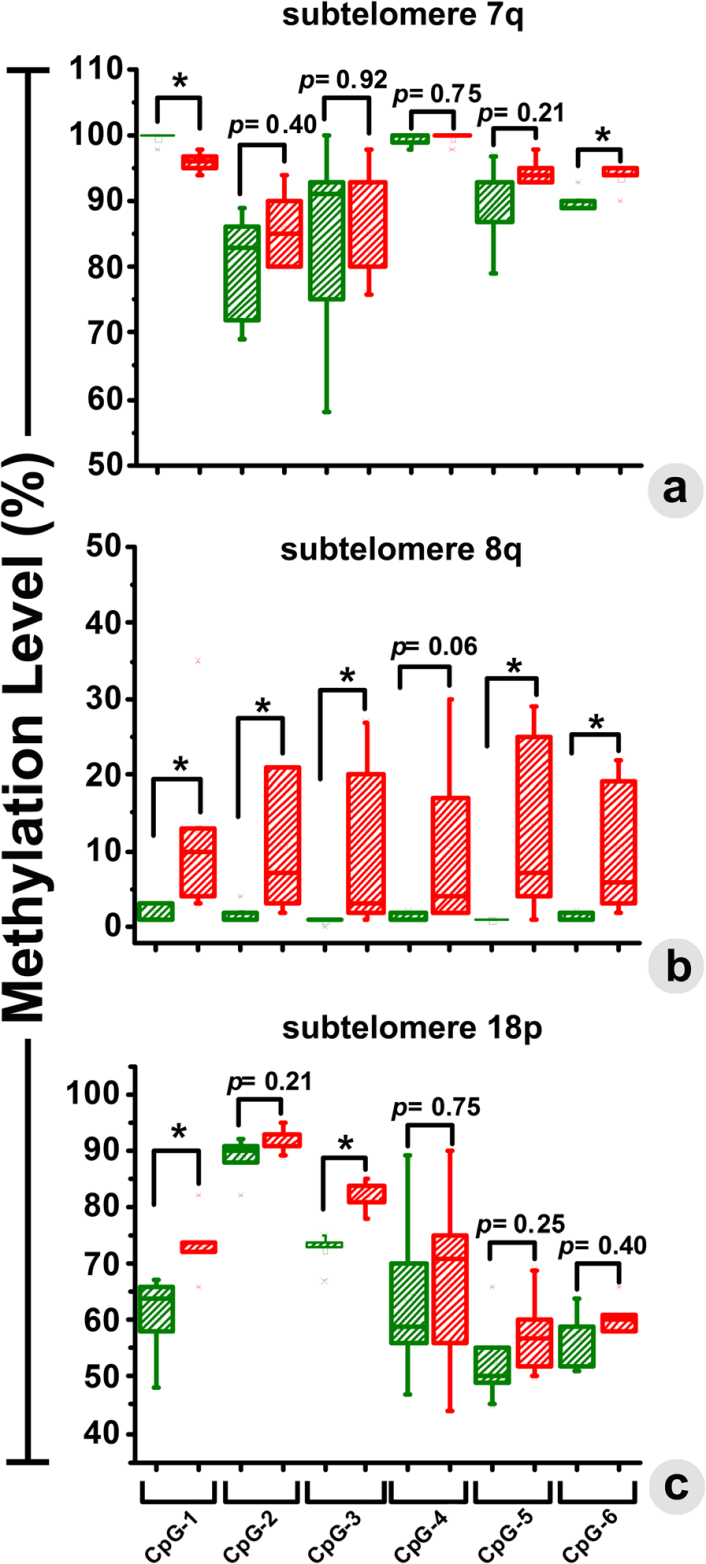
The changes in methylation level (%) were quantitatively determined at each of the CpG sites by pyrosequencing. Significant changes in the percentage of methylation were observed in two out of six CpGs in Chr. 7q (**a**), five out of six CpGs in Chr. 8q (**b**), and two out of six CpG sites in Chr. 18p (**c**). Methylation in glioma and control patients is presented with *green* and *red* colored box-plots, respectively

**Fig. 4:**
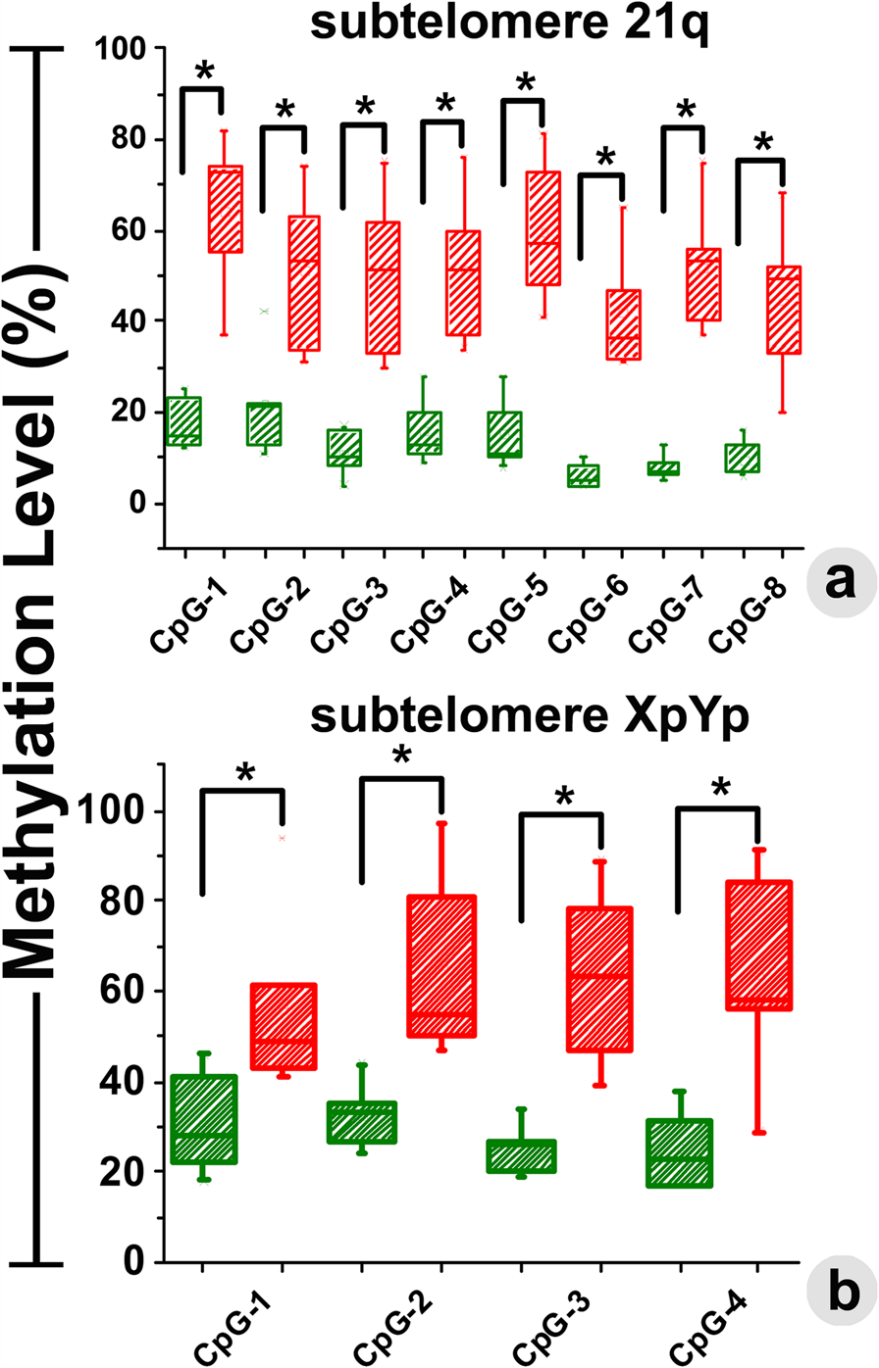
The changes in methylation level (%) were quantitatively determined at each of the CpG sites in Chr. 21q (**a**) and Chr. XpYp (**b**). In both the chromosomes, we have observed significant increase in methylation level among glioma patients in comparison to the group of control patients. Methylation in glioma and control patients is presented with *green* and *red* colored box-plots, respectively

The observed changes in methylation through MSP were cross-validated by quantitatively determining the percentage of methylation at each CpGs of the observed subtelomeric sites, using pyrosequencing analysis. The difference in the mean methylation at Chr. 7q subtelomere between the non-glioma (90.34 ± 5.50 %) and glioma patients (92.5 ± 1.72 %) was found to be insignificant (*p* = 0.67) (Fig. 2f), which was consistent to the MSP data. Moreover, no significant change in methylation was observed in four out of six CpG sites (except for CpG1 and CpG6) between the non-glioma and glioma patients (Fig.3a). A conflicting result was obtained from MSP and pyrosequencing analyses relating to the mean methylation level at Chr.8q subtelomere. MSP data showed insignificant (*p* = 0.08) difference in the average mean methylation level between the groups, while the pyrosequencing analysis revealed the change in mean methylation level from 1.4 ± 0.57 % to 11.5 ± 2.25 % between the control and glioma patients, which was significant (*p* < 0.05) (Fig. 2g). Nonetheless, five of the six CpG sites were significantly methylated in the glioma group (Fig.3b). The change in methylation at five CpG sites hence makes this region a suitable candidate as a biomarker of the disease. The change in methylation level between the test groups at the Chr. 18p subtelomere also showed disparity between MSP and pyrosequencing as the Chr. 8q. The mean methylation difference between the non-glioma (66 ± 5.62 %) and glioma groups (72.2 ± 3.53 %) were statistically insignificant (*p* = 0.09), as obtained from the pyrosequencing (Fig. 2h), which was inconsistent with the results from MSP (Fig. 2c). Two of the six CpGs (CpG1 and CpG3) in 18p subtelomere were significantly methylated only in glioma patients (Fig.3c). In contrast, the mean methylation level from non-glioma to glioma patients changed from 13.25 ± 4.7 % to 51.97 ± 15.45 % in chromosome 21q (Fig. 2i) and from 28.5 ± 8.29 % to 62.7 ± 20.34 % in chromosome XpYp (Fig. 2j), both of which were considered to have a significant difference in methylation. Moreover, all of the CpG sites at these two chromosomes were significantly methylated in glioma patient groups (Fig.4a, b). Based on these findings, it can be summarized that selective increase in subtelomeric methylation at Chr. 8q, Chr.21q and Chr. XpYp could serve as potential epigenetic biomarkers of glioma.

### Correlation between subtelomeric methylation and telomere length

**Fig. 5:**
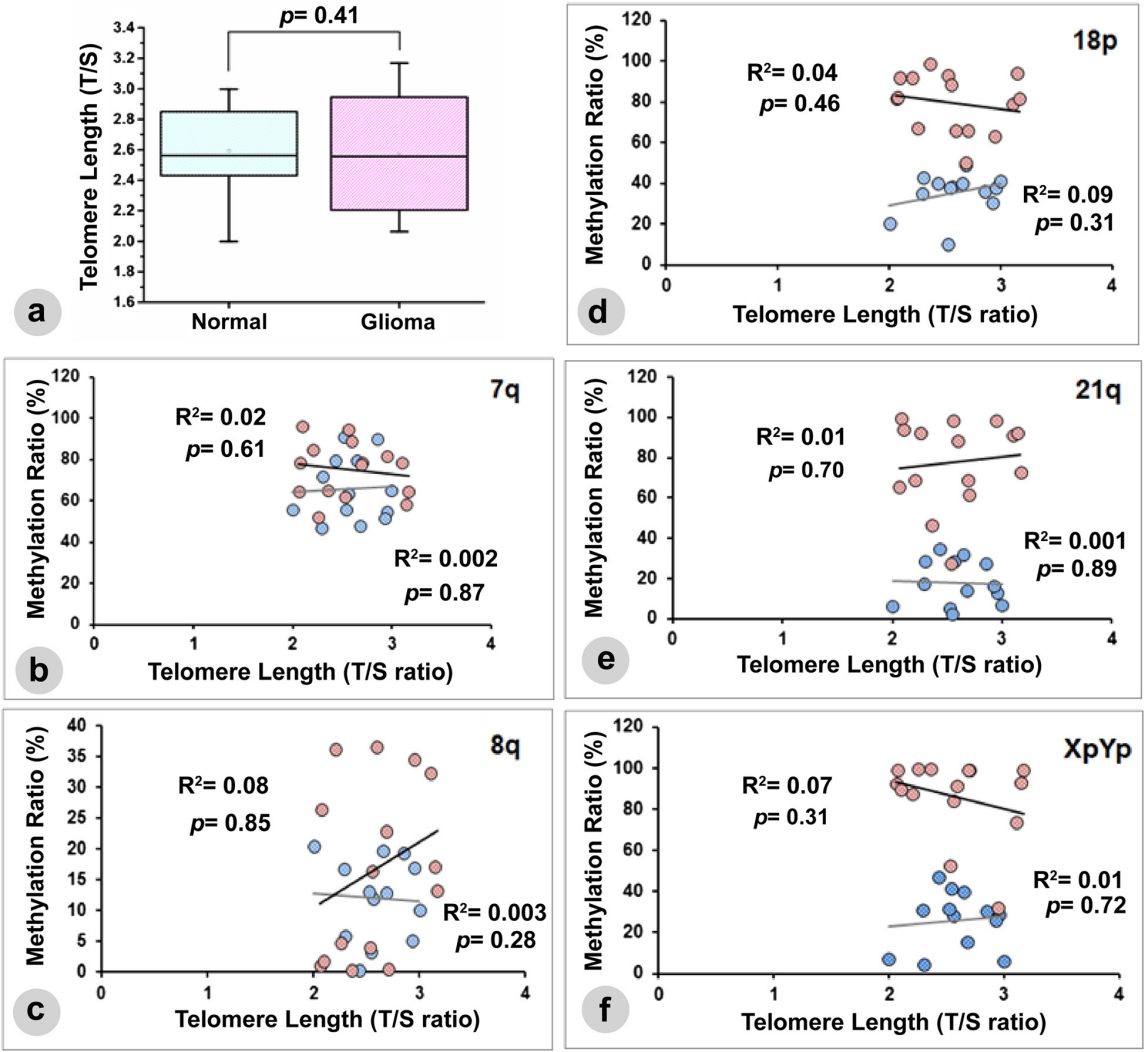
Relationship between subtelomeric methylation status and telomere length in glioma. **a** Relative telomere length (T/S), expressed as the relative ratio of the copy number of telomeric repeats to a single copy gene 36B4, in both control and glioma patients. Correlation between telomere length (TL) and subtelomeric methylation ratio (as obtained from MSP) at five chromosomal ends was evaluated in both the control and glioma patients (**b**–**f**). Methylations in glioma and control patients are presented with *pink dots* and *blue dots*, respectively, while the resulting linear lines correspond to each group were presented by a *black* and *gray lines*, respectively. We did not observe any significant correlation between the methylation ratios to the global change in TL

The relative TL (T/S) ranged from 6.17 to 7.71 kb, with a mean of 7.0876 ± 0.45 kb in non-glioma patients, and ranged from 6.27 to 7.96 kb, with the mean of 7.14 ± 0.57 kb in glioma patients (Fig. 5a). The mean change in relative TL was not significantly different (*p* = 0.41) between the test groups. Simultaneously, we have also determined the correlation between the subtelomeric methylation level (%) at five chromosome ends to the change in global TL in both the control and glioma patients. However, no linear correlation was established between these two parameters. Moreover, the correlations between the methylation levels from individuals in each group to the corresponding TLs were also not significant (Fig. 5b, f). Based on these observations, we hypothesize that changes in subtelomeric methylation do not influence TL in glioma.

### Induced demethylation and its correlation to the telomere length

**Fig. 6:**
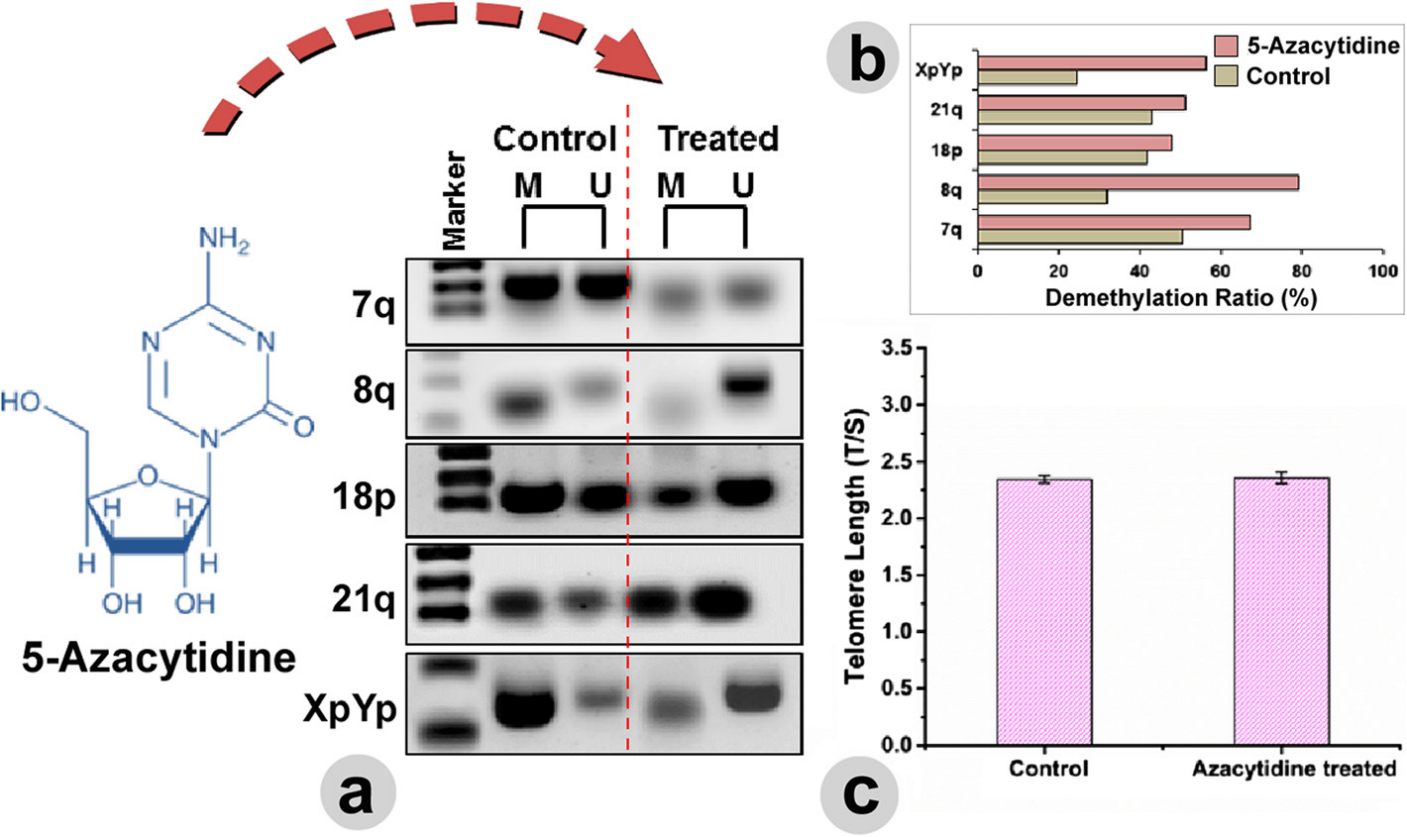
**a** Induced demethylation was recognized with the increased band intensity of the amplicons against the unmethylation (U)-specific primers in AZA-treated SF-767 cell line. **b** The adjacent bar chart, extracted from the gel image expresses the change in demethylation ratio between the non-treated and AZA-treated replica. The demethylation ratio at each of the subtelomeres was increased in comparison to the untreated replica. Nonetheless, we did not observe any significant alteration in TL after treatment with AZA (**c**).

To cross validate the lack of correlation between TL and subtelomeric methylation in glioma, a global demethylation was induced in SF-767 cells with AZA. Subtelomeric methylation level at each of the chromosomes was then determined alongside the global TL, before and after the drug treatment. The band intensity of the demethylation specific amplicons from the subtelomeric regions of each chromosome was found to increase upon drug treatment (Fig.6a). We have also determined the change in demethylation ratio among the AZA-treated genomic replicates over the negative control (Fig.6b). The highest increment in the demethylation ratio was observed for chromosome 8q, followed by XpYp, 7q, 21q, and 18p. However, we have noticed no significant change in the TL (T/S) between the untreated (2.346 ± 0.034) and AZA-treated (2.358 ± 0.049) cells (Fig. 6c). These lines of experiments served as the in vitro validation of the observed phenomenon in the clinical isolates and also support the claim that there may not be any significant correlation between subtelomeric DNA methylation level and global change in TL in glioma.

## Discussion

Majority of cancer cells utilize telomerase to maintain the TL through the recurrent recruitment of TTAGGG repeat sequences at the chromosome ends [33]. Telomerase is the ribonucleoprotein enzyme responsible for DNA polymerization at telomeres and helps to overcome the end-replication problems associated with progressive cell divisions [34]. However, a subset of tumors including malignant glioma in adults utilize alternative lengthening of telomeres (ALT) to sustain their self-renewal activity, which involves recombination-mediated replication of telomeric DNA [35, 36]. The occurrence of subtelomeric DNA hypomethylation is often defined as a favorable inducer of ALT [37]. However, a plethora of reports are available, which do not support this claim [38, 39]. It was found that instead of loss in methylation, subtelomeres may acquire dense methylation in some specific cell lines such as in SK-LU-1, towards telomere lengthening [21]. These cumulative findings strongly suggest that subtelomeric DNA methylation pattern might vary from one cell line to the other and also varies in different type of cancers, and hence, can be rationally used as epigenetic biomarker of specific malignancies [21, 27, 40]. Previously, variation in methylation pattern for the same subtelomeric regions was reported in regard to hepatocellular carcinoma [27]. The present study reports the selective changes in methylation level at these subtelomeric CpG sites for malignant glioma.

From our results, we observed that the subtelomere of Chr.7q was hypermethylated, whereas hypomethylation was observed at Chr. 8q, 18p, 21q, and XpYp subtelomeres in control patients. Nonetheless, significant increase in subtelomeric methylation level was observed for all the chromosomes in the glioma patients. The significant change in the methylation level during gliomagenesis was noticed at all age groups compared to control patients, which also makes these CpG sites good biomarker candidates for early detection of disease. We cross-validated the MSP results with the change in subtelomeric methylation between the groups using pyrosequencing. Herein, we observed that except for Chr. 8q and 18p, both the assays were in agreement with each other for the observed regions in all the chromosomes. In this regard, we would like to consider the pyrosequencing data as methylation determinant in Chr. 8q, and 18p, since false positive results are often obtained in MSP due to mis-priming, high number of replication cycle, or subtle difference in DNA concentration [41]. Moreover, in pyrosequencing analysis, we can cover many more CpG islands than MSP. This might result in significant difference between the final outcomes between these two methods, irrespective of the fact that pyrosequencing primers flanked the MSP sites. Based on these observations, we consider that selective increase in methylation level, identified at Chr.8q, 21q, and XpYp subtelomeres, may be used as strong and unequivocal epigenetic marker of glioma. However, we did not observe any significant difference in the genome-wide 5-mC % between the control and diseased group. This also supports the relevance of using selective changes in the subtelomeric methylation as the biomarker of the disease.

We have also evaluated the possible effect of altered subtelomeric methylation on the telomere lengthening, but did not find any significant correlation. In fact, there was no remarkable change in TL between the control and glioma patients. These results corroborate with the previous finding, where no detectable changes were observed in TL or no correlation between TL and the risk of glioma was established [42]. In addition, induced demethylation in SF767 cells followed by AZA treatment resulted in insignificant changes in the TL but significant alterations in the subtelomeric methylation profile. This confirmed that there may not be a direct correlation between the subtelomeric methylation and telomere lengthening in glioma. We believe that this needs whole-genome DNA methylation sequencing of individual subtelomeres at each chromosome, which should be compared with the corresponding length of the telomere. Although the present study reports the selective increase in subtelomeric methylation as an epigenetic biomarker of glioma, the major impasse for implementing this screening method could lie with the limited access for brain biopsies in patients. However, we believe that if the present screening technique could be coupled with novel tools such as circulating tumor cell collection method [43], it could serve as the non-invasive platform for the detection of glioma in patients. Finally, since the increase in selective subtelomeric methylation level was observed in patients even with low-grade glioma, this method can also aid in the early detection of the disease.

## Conclusions

Methylation level significantly and invariably increased at the subtelomeres of Chr. 8q, 21q, and XpYp in patients with different grades of glioma. The changes in methylation level are well demonstrated by both the MSP and pyrosequencing data and are independent of demographic and clinicopathologic features of the patients. Changes in methylation levels at the subtelomeric sites among glioma patients hence could be proposed as potential epigenetic biomarkers of the disease regardless of their insignificant correlation with TL. The overall findings of our study also provide a solid platform to further investigate the additional epigenetic interplay at the subtelomeric loci during diseased state.

## Additional file

Additional file 1:
**Supplementary information.** This contains Tables S1–S2, Figures S1–S3, and supplementary note.

## Abbreviations

5-mC: 5-methylcytosine
ALT: alternative lengthening of telomeres
AZA: 5-azacytidine
Chr: chromosome
M: methylation
MGMT: O^6^-methylguanine DNA-methyltransferase
MSP: methylation specific polymerase chain reaction
T/S: relative telomere length
TL: telomere length
TERRA: telomere repeat-containing RNA
U: unmethylation
