# Supplementary material for: Selective increase in subtelomeric DNA methylation: an epigenetic biomarker for malignant glioma
Source: Clin Epigenetics. 2015 Oct 7;7:107. doi: 10.1186/s13148-015-0140-y (PMC4597615; doi:10.1186/s13148-015-0140-y)
Supplement: Additional file 1: — Supplementary information. This contains Tables S1–S2, Figures S1–S3, and supplementary note. [file 13148_2015_140_MOESM1_ESM.doc]

**Supplementary Information**

**Selective increase in subtelomeric DNA methylation: an epigenetic biomarker for malignant-glioma**

Samrat Roy Choudhury^1^, Yi Cui^1^, Jacob R Milton^2^, Jian Li^3^, Joseph Irudayaraj^1*^

^1^Department of Biological Engineering, Center for Cancer Research, Purdue University, West Lafayette, IN 47906, USA, ^2^Department of Biological Sciences, Purdue University, West Lafayette, IN 47906, USA, ^3^Department of Neurosurgery, Xiangya Hospital, Central South University, Changsha, Hunan 410008, China

^*^Corresponding author

Tel: +1 (765) 494-0388, Fax: +1 (765) 496-1115

Email: [josephi@purdue.edu](mailto:josephi@purdue.edu)

**Table-S1**: Primer sequences used for bisulfite PCR (BSP) in 5’ to 3’ direction.

| **Subtelomeric region** | **Forward primer sequence** | **Reverse primer sequence** | **Product size (bp)** | **Annealing temperature (°C)** |
| --- | --- | --- | --- | --- |
| **7q** | AGTGGATATTTAGGTTTATGATTGTAAT | **5’-/5BiosG/**CTCCCCAAAAAACACTTTACTCTTTCACA | 257 | 58 |
| **8q** | TGTTTTTTTTTTTTTATTAAAAGGTGAGTT | **5’-/5BiosG/**AAAAATCTCCAAAAAACCCTTATAAAC | 139 | 57 |
| **18p** | **5’-/5BiosG/**GTTGTGGTGTAGGTTTATAATTT | ACCCCCAAAAAAAAACTACAAAAACC | 185 | 56 |
| **21q** | TTTTGGAGGATTGTGTTTAGATAGATAT | **5’-/5BiosG/**CCCAACTCCTTCCAAATAAAT | 137 | 58 |
| **XpYp** | GTGGGTTAGTTTGTATTGAGGTAGGTAT | **5’-/5BiosG/**AAAACCCCCTAACCCCTCC | 307 | 57* |

**5’-/5BiosG/** is the 5'-biotinylation on the forward or reverse BSP primers. **^*^**Xp subtelomere region was amplified in presence of Q-solution (QIAGEN).

**Table-S2**: Analyzed sequence and the sequencing primers for the pyro-sequencing assay; all primers listed are 5' to 3'.

| **Subtelomeric region** | **Sequenced CpG region** | **Primer sequence** |
| --- | --- | --- |
| **7q** | AAATTTGTTATYGTAGTGGTYGGATTTTTTTGGGTTATTYGAGTGGAGYGGTYGGGTT | TTTATGATTGTAATTTTATAATA |
| **8q** | YGGGTAYGTAGTTAGYGTTYGYGATTATTYGTTTATAAGGGTTTTTT | ATTTTTATTAGGAAAGGTGT |
| **18p** | AACCRCCCTCRCAACCCAAATAACCRATTTATCCTACCCRAAAAACATCR CCCTCCTCCRCCRAACCCRAACCC | CTAAACCCCCCCTTCTACCCCTA |
| **21q** | TTYGTAGGTGTGYGYGGYGTTYGTAAGTGGTTAGTATAAYGTYGGGYGAA | GTTTAGATAGATATTGGTTTTTTTA |
| **XpYp** | TYGAGGTGATTYGGGTGGGAGGTGTATYGTYGTTTATT | GAGGTAGGTATGGGT |

Y/ R (complement of Y) are the potential methylated cytosines at the CpG sites.

**
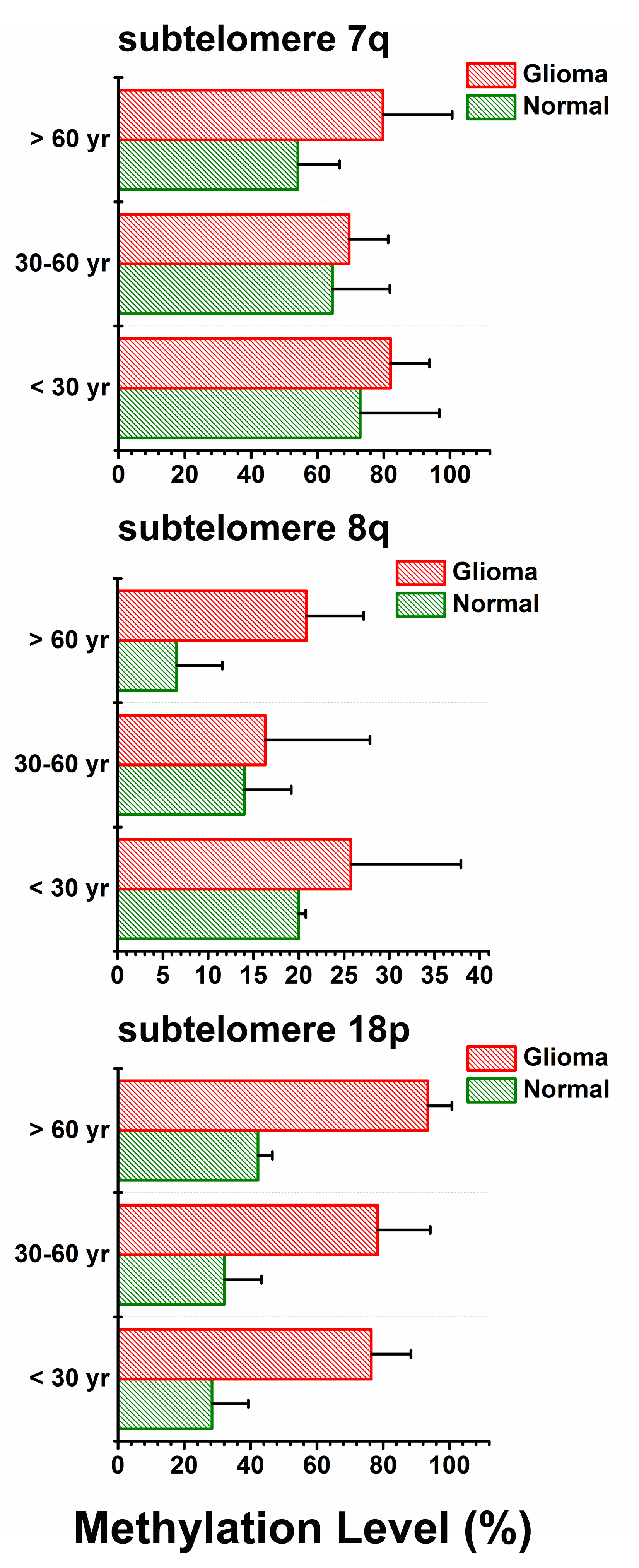
**

**Figure S1. Difference in methylation level between control and glioma patients at different age groups:** Both the control (n= 13) and glioma affected individuals (n= 15) were broadly categorized into 3 age groups, namely, <30 yrs., 30-60 yrs., and > 60 yrs. A gradual reduction in methylation level (%) was observed in chromosome 7q and 8q subtelomeres along the increase in age among the control patients. In contrast, methylation level remained significantly high at the same subtelomeric sites among the glioma patients. Chr. 18p subtelomere showed slight increase in methylation for the control patients over the age. On contrary, Chr. 18p subtelomere showed higher level of methylation in all the age groups compared to the control patients. The methylation level also showed an incremental trend over the ages among the glioma patients.


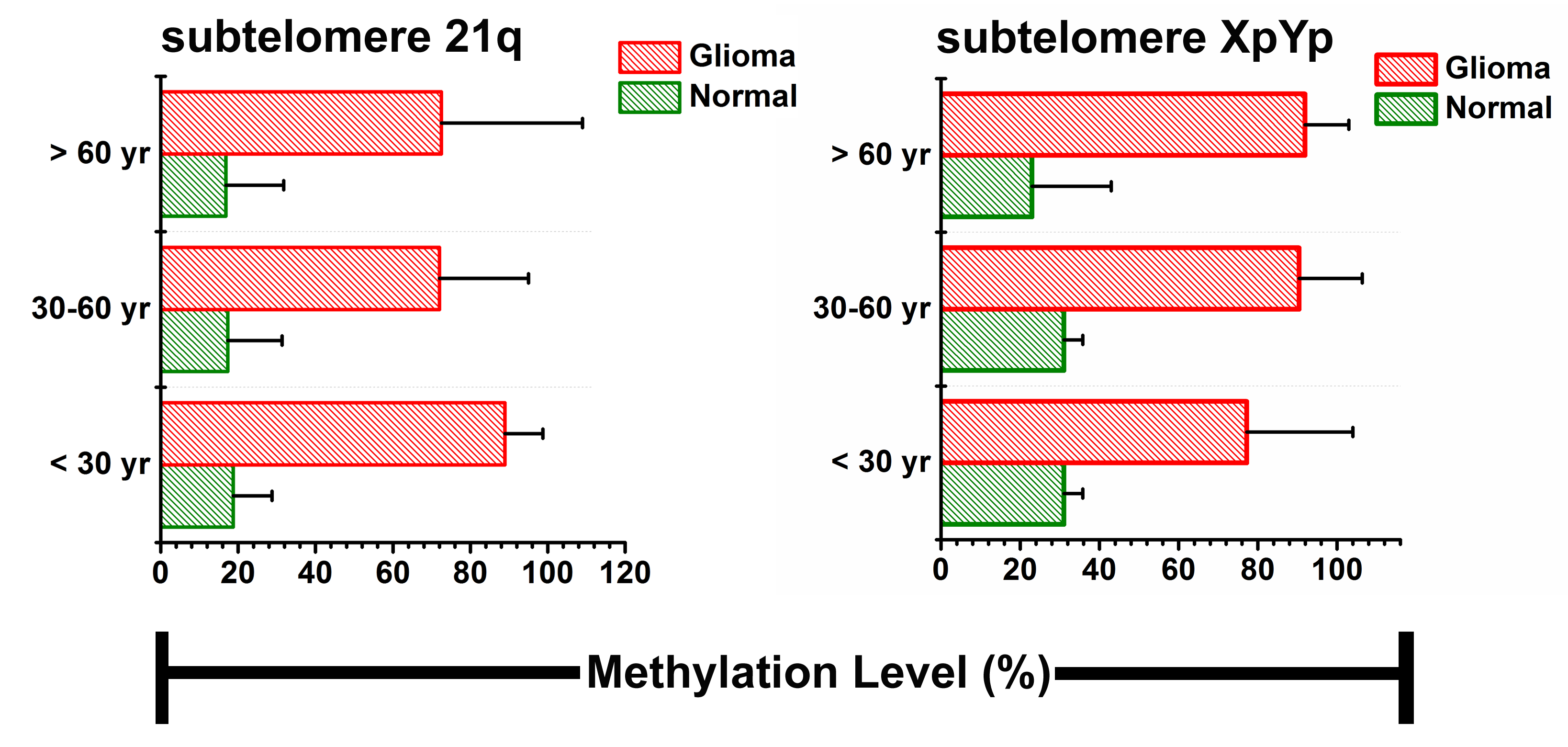


**Figure S2. Difference in methylation level between control and glioma patients at different age groups.** Both the control (n= 13) and glioma affected individuals (n= 15) were broadly categorized into 3 age groups, namely, <30 yrs., 30-60 yrs., and > 60 yrs. The subtelomeric methylation level (%) remained almost same at different age groups in Chr. 21q among control patients. In contrast, methylation level was significantly higher in glioma patients at all age groups. Glioma patients also showed significantly higher level of methylation at all ages at the subtelomere of Chr. XpYp in comparison to the control patients.


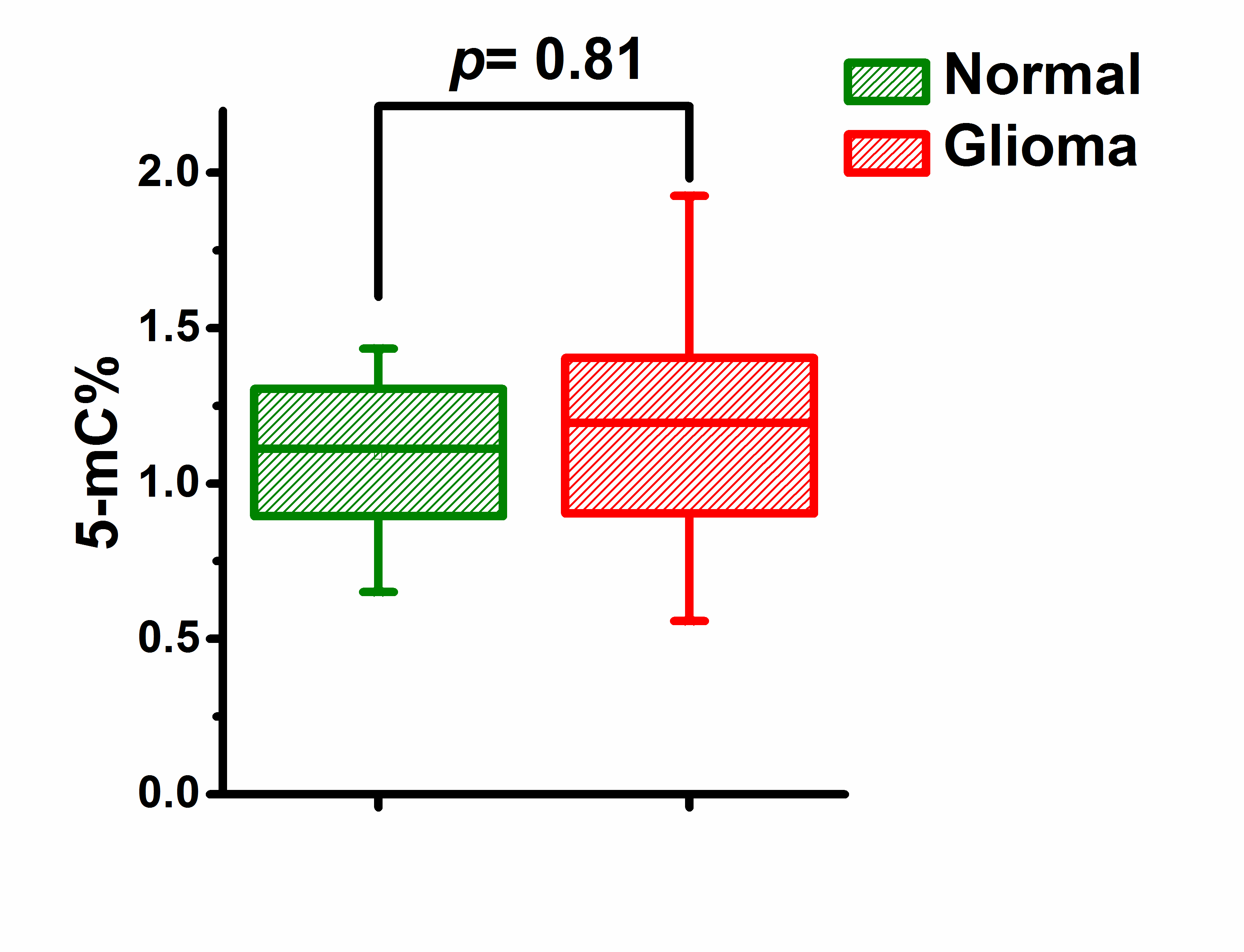


**Figure S3.** The global methylation, represented as a percentage of 5-methylcytosine (5-mC %) in control and glioma patients was determined in triplicate. The coefficient of variation (*Cv*) between the three round of experiments varied from 0.199 to 0.258 in control (n=13) and from 0.396 to 0.403 in glioma (n=15) affected group. No significant change in 5-mC % was noted between the groups.

**Supplementary note**

The range of coefficient of variation (Cv) between the independent rounds of experiments are summarized as follows:

**Average change in methylation ratio, as obtained from MSP:**

***Chr. 7q:*** The Cv in control patients ranged between 0.2 and 0.23, while in glioma patients it was in the range between 0.16 and 0.1; ***Chr. 8q:*** The Cv in control patients ranged between 0.51 and 0.55, while in glioma patients it was in the range between 0.49 and 0.57; ***Chr.18p:*** The Cv in control patients ranged between 0.28 and 0.31, while in glioma patients it was in the range between 0.15 and 0.17; ***Chr. 21q:*** The Cv in control patients ranged between 0.51 and 0.55, while in glioma patients it was in the range between 0.49 and 0.57; ***Chr. XpYp:*** The Cv in control patients ranged between 0.52 and 0.53, while in glioma patients it was in the range between 0.2 and 0.22.

**Average change in methylation ratio, as obtained from pyrosequencing:**

***Chr. 7q:*** The Cv in control patients was in the range between 0.05 and 0.06, while in glioma patients it was between 0.10 and 0.11; ***Chr. 8q:*** The Cv in control patients was in the range between 0.401 and 0.403, while in glioma patients it was between 0.37 and 0.379; ***Chr.18p:*** The Cv in control patients was in the range between 0.081 and 0.086, while in glioma patients it was between 0.049 and 0.05; ***Chr. 21q:*** The Cv in control patients was in the range between 0.355 and 0.358, while in glioma patients it was between 0.297 and 0.31; ***Chr. XpYp:*** The Cv in control patients was in the range between 0.291 and 0. 3, while in glioma patients it was between 0.324 and 0.325.
